# Supplementary material for: The Influence of Strain and Sex on High Fat Diet-Associated Alterations of Dopamine Neurochemistry in Mice
Source: Nutrients. 2024 Sep 29;16(19):3301. doi: 10.3390/nu16193301 (PMC11479034; doi:10.3390/nu16193301)
Supplement: Supplementary file 1 [file nutrients-16-03301-s001.zip › Nutrients Revision_Supplementary Table 1.pdf]

Table 1: Nutrient composition of assigned diets.

**Grams and kilocalories by ingredient**					
Class Description	Ingredient	**CFD** Research Diets, Inc. D12450		**HFD** Research Diets, Inc. D12492	
		Grams	Kilocalories	Grams	Kilocalories
Protein	Casein, Lactic, 30 mesh	200.00	800.00	200.00	800.00
Protein	L-Cysteine	3.00	12.00	3.00	12.00
Carbohydrate	Sucrose, Fine Granulated	72.80	291.20	72.80	291.20
Carbohydrate	Starch, Corn	506.20	2024.80	-	-
Carbohydrate	Lodex 10	125.00	500.00	125.00	500.00
Fiber	Solka Floc, FCC200	50.00	200.00	50.00	200.00
Fat	Soybean Oil, USP	25.00	225.00	25.00	225.00
Fat	Lard	20.00	180.00	245.00	2205.00
Mineral	Mineral Mix, S10026B	50.00	-	50.00	-
Vitamin	Choline bitartrate	2.00	-	2.00	-
Vitamin	Vitamin Mix, V10001C	1.00	-	1.00	-
Dye	Yellow dye FD&C #5	0.04	-	-	-
Dye	Blue dye FD&C #1	0.01	-	0.05	-
Total		1055.05	4233.00	773.85	4233.20
**Percent of kilocalories by macronutrient**					
Protein			20%		20%
Carbohydrate			70%		20%
Fat			10%		60%
