# Supplementary figures and images for: The Influence of Strain and Sex on High Fat Diet-Associated Alterations of Dopamine Neurochemistry in Mice
Source: Nutrients. 2024 Sep 29;16(19):3301. doi: 10.3390/nu16193301 (PMC11479034; doi:10.3390/nu16193301)

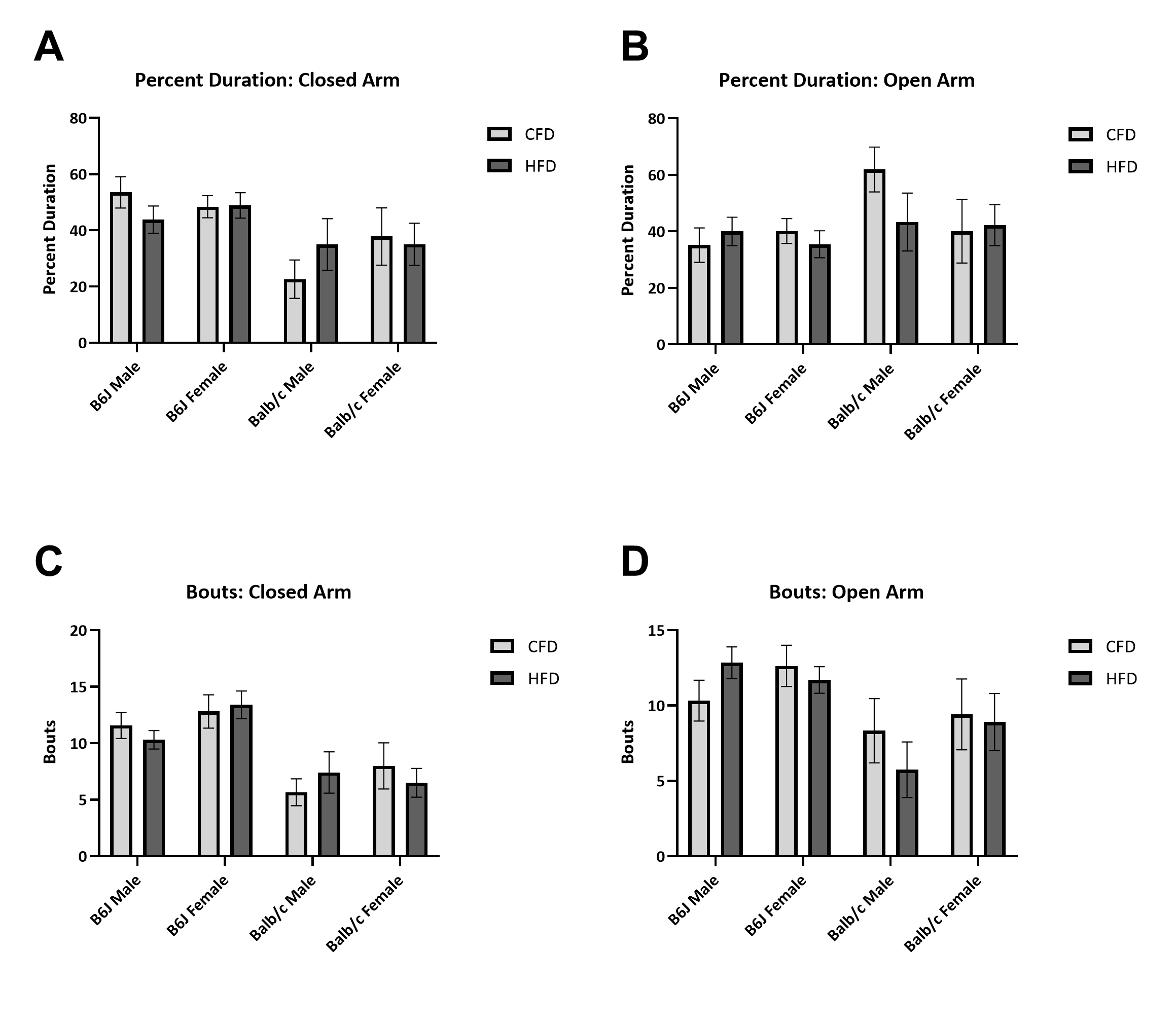

Supplement: Supplementary file 1 [file nutrients-16-03301-s001.zip › Supplementary Figure 1.tif]

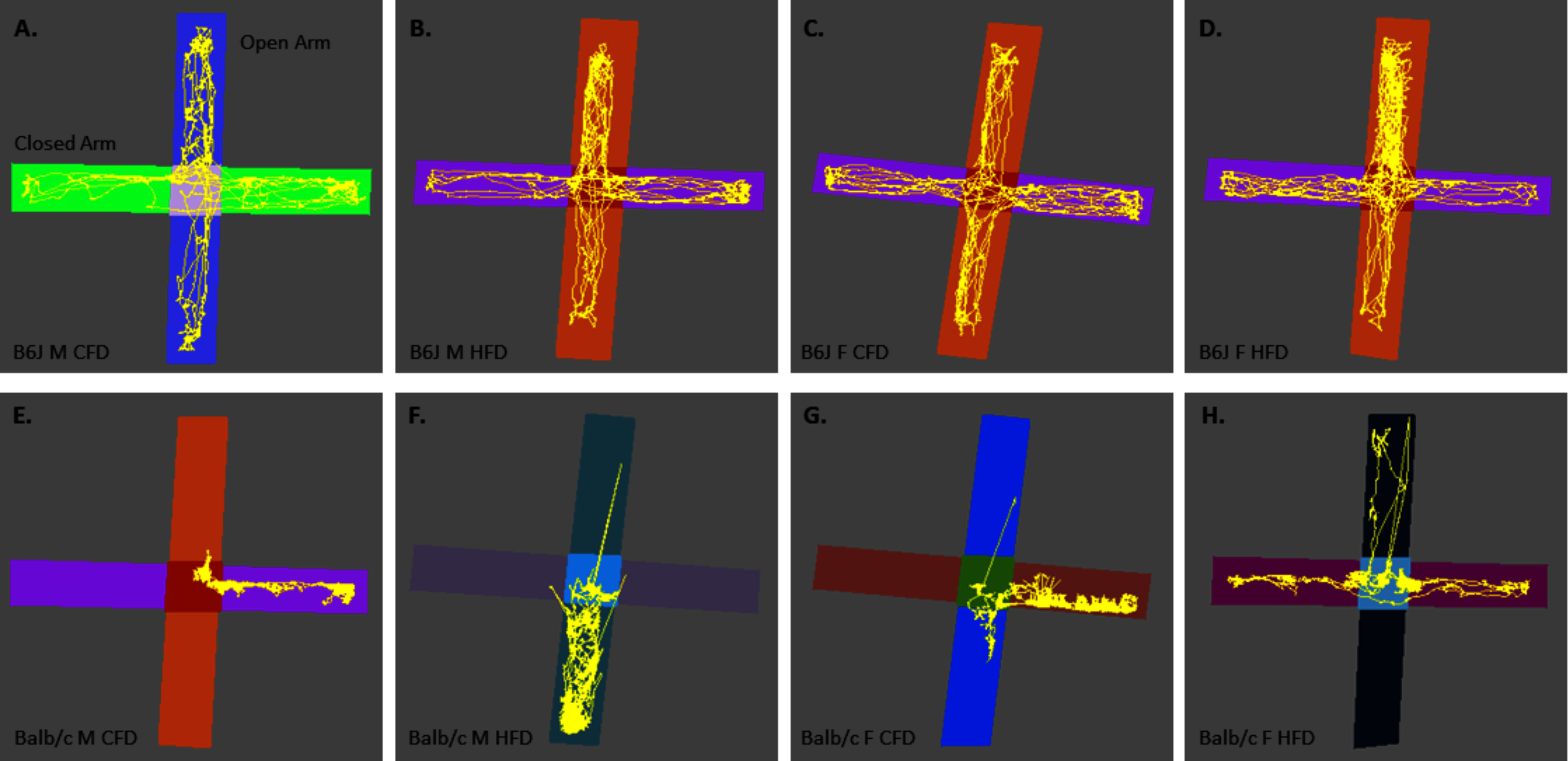

Supplement: Supplementary file 1 [file nutrients-16-03301-s001.zip › Supplementary Figure 2.jpg]

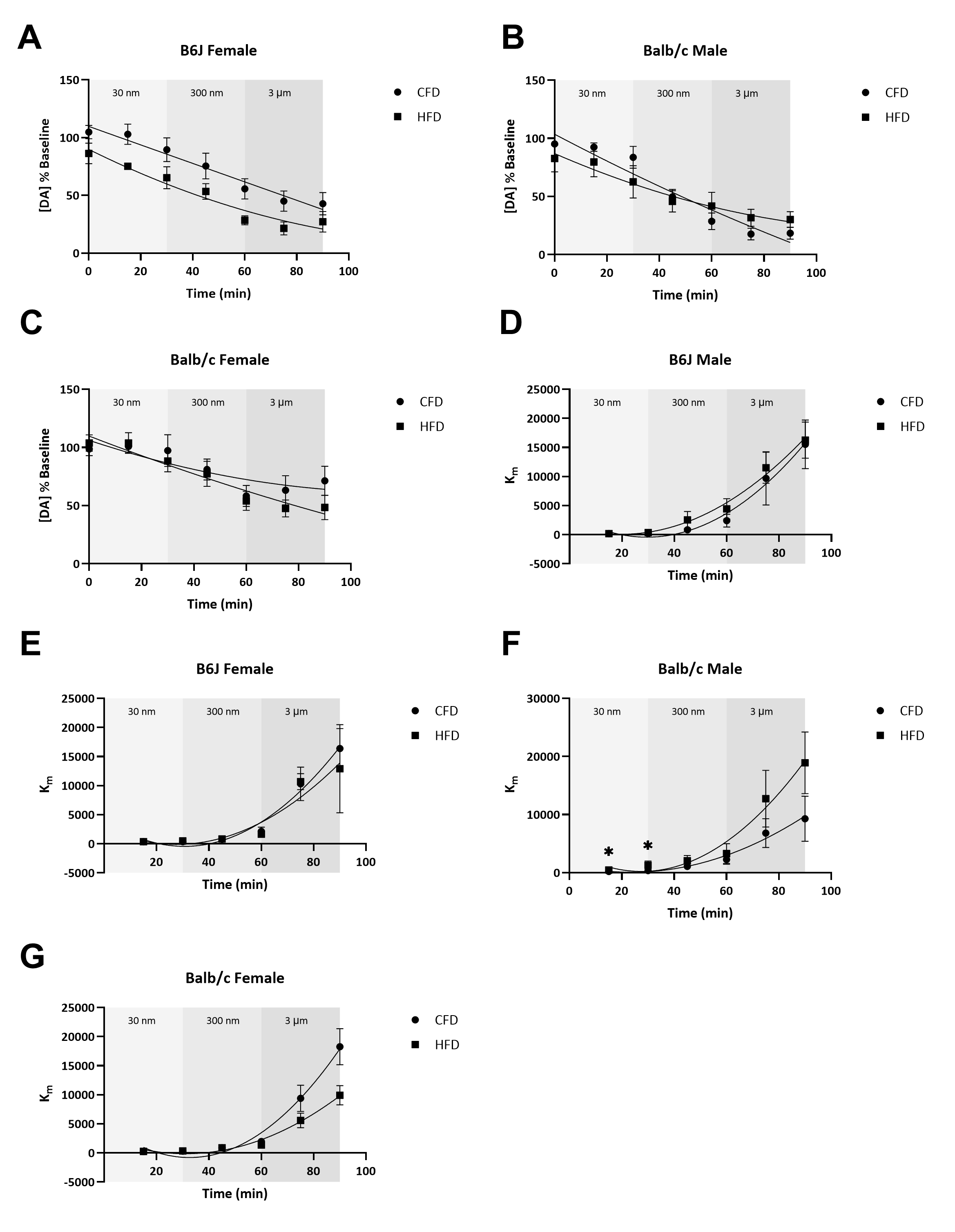

Supplement: Supplementary file 1 [file nutrients-16-03301-s001.zip › Supplementary Figure 3.png]
